# Retracted: Why Fish Oil Fails: A Comprehensive 21st Century Lipids-Based Physiologic Analysis

**DOI:** 10.1155/2014/832729

**Published:** 2014-11-05

**Authors:** Journal of Lipids

The article titled “*Why Fish Oil Fails: A Comprehensive 21st Century Lipids-Based Physiologic Analysis*” [1], published in Journal of Lipids has been retracted as a result of an undeclared competing interest on the part of the manuscript's author.
